# Pulmonary Thromboembolism at High Altitude in a Previously Healthy Adult: A Case Report

**DOI:** 10.7759/cureus.83001

**Published:** 2025-04-25

**Authors:** Rohini Kumari, Nidhi Kaeley, Aditya S Mahalle, Parina Tejpal, Sreevardhan Ambati

**Affiliations:** 1 Emergency Medicine, All India Institute of Medical Sciences, Rishikesh, IND

**Keywords:** acute pulmonary embolism, hape, high altitude, high-altitude, perc criteria, sudden-onset breathlessness

## Abstract

Travel to high altitudes presents unique physiological challenges and can lead to various acute illnesses. Although pulmonary thromboembolism is infrequently documented in these settings, it can be life-threatening if unrecognized. A 54-year-old man with no known comorbidities developed sudden-onset shortness of breath and altered mental status while traveling at high altitudes. Initial evaluation revealed hypoxia, fever, and a markedly reduced ejection fraction on point-of-care ultrasound, with no deep vein thrombosis (DVT) on Doppler studies. Further imaging identified minimal pleural effusion and atelectasis on high-resolution computed tomography of the thorax. Despite a low clinical probability based on scoring systems and a negative D-dimer, clinical suspicion prompted a computed tomographic pulmonary angiography, which confirmed pulmonary thromboembolism in the right lower lobe pulmonary artery. Treatment with apixaban led to symptomatic improvement, and the patient was discharged in stable condition. This case highlights the need to consider pulmonary thromboembolism in the differential diagnosis of respiratory distress at high altitude, where hypoxia and other altitude-related factors may contribute to a hypercoagulable state.

## Introduction

Many people travel to mountainous areas worldwide each year, and high-altitude illness is common in this population due to physiological changes at higher elevations [1]. In Uttarakhand, India, pilgrims travel annually for the Chardham Yatra (a religious pilgrimage to four Hindu holy sites), but not all individuals are medically fit to ascend. Not everyone successfully adapts to the high-altitude environment even among those who are fit. Consequently, many cases of high-altitude pulmonary edema (HAPE) and high-altitude cerebral edema are encountered during the pilgrimage season.

According to Marx et al., the incidence of HAPE varies from 0.01% to 2%, but it has reached 15.5% among soldiers who were flown directly to an elevation of 14,500 ft [1]. However, pulmonary thromboembolism is rarer in high-altitude settings [2], although such presentations have been encountered in the Emergency Department at All India Institute of Medical Sciences, Rishikesh. Pulmonary thromboembolism is a life-threatening condition that must be identified early and treated accordingly. Risk factors for pulmonary embolism (PE) include deep vein thrombosis (DVT), inherited or acquired thrombophilia, recent surgery or immobilization, cancer, and smoking. Nonetheless, PE at high altitudes remains very rare, with few cases published to date, and few studies have indicated that HA is a potential risk factor for PE [3]. Here, we present one such case of a 54-year-old man who arrived at the Emergency Department in May 2024 during the Chardham Yatra.

## Case presentation

A 54-year-old man from West Bengal with no known comorbidities arrived in Haridwar on 12 May 2024 to begin the Chardham Yatra. He traveled to Barkot (13 May), Yamunotri (3291 m, 14 May), Gangotri (3415 m, 16 May), and Kedarnath (3553 m, 18 May). On 19 May, he developed a sudden-onset shortness of breath and altered mental status that had been present for one day. He also reported a four-day history of low-grade fever (undocumented, intermittent, and relieved by medications, with no diurnal variation) and a five-day history of cough with sputum production.

**Figure 1:**
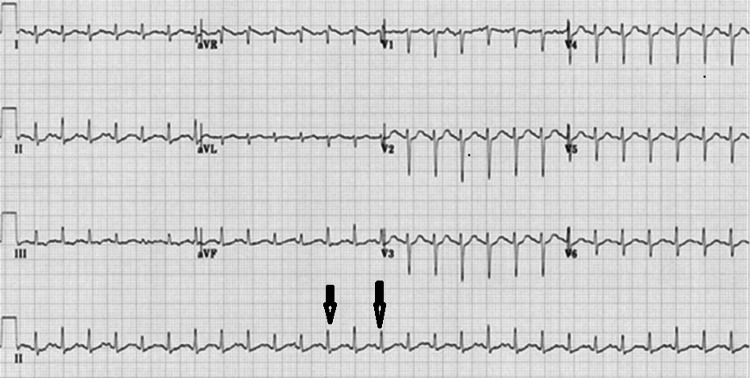
Electrocardiogram showing sinus tachycardia (marked by black arrow)

Upon arrival at our hospital, the patient had a patent airway, a respiratory rate of 28 breaths/min, oxygen saturation of 70% on room air, a pulse rate of 121 beats/min, blood pressure of 130/78 mmHg, and a temperature of 102°F. The primary survey was completed, and he was started on 4 L of supplemental oxygen via nasal prongs. The electrocardiogram (Figure 1) showed sinus tachycardia. Point-of-care ultrasound revealed an ejection fraction of 20%, with no right atrium or right ventricle dilation and no regional wall motion abnormality. A bilateral “A-profile” was noted in all lung zones.

**Figure 2:**
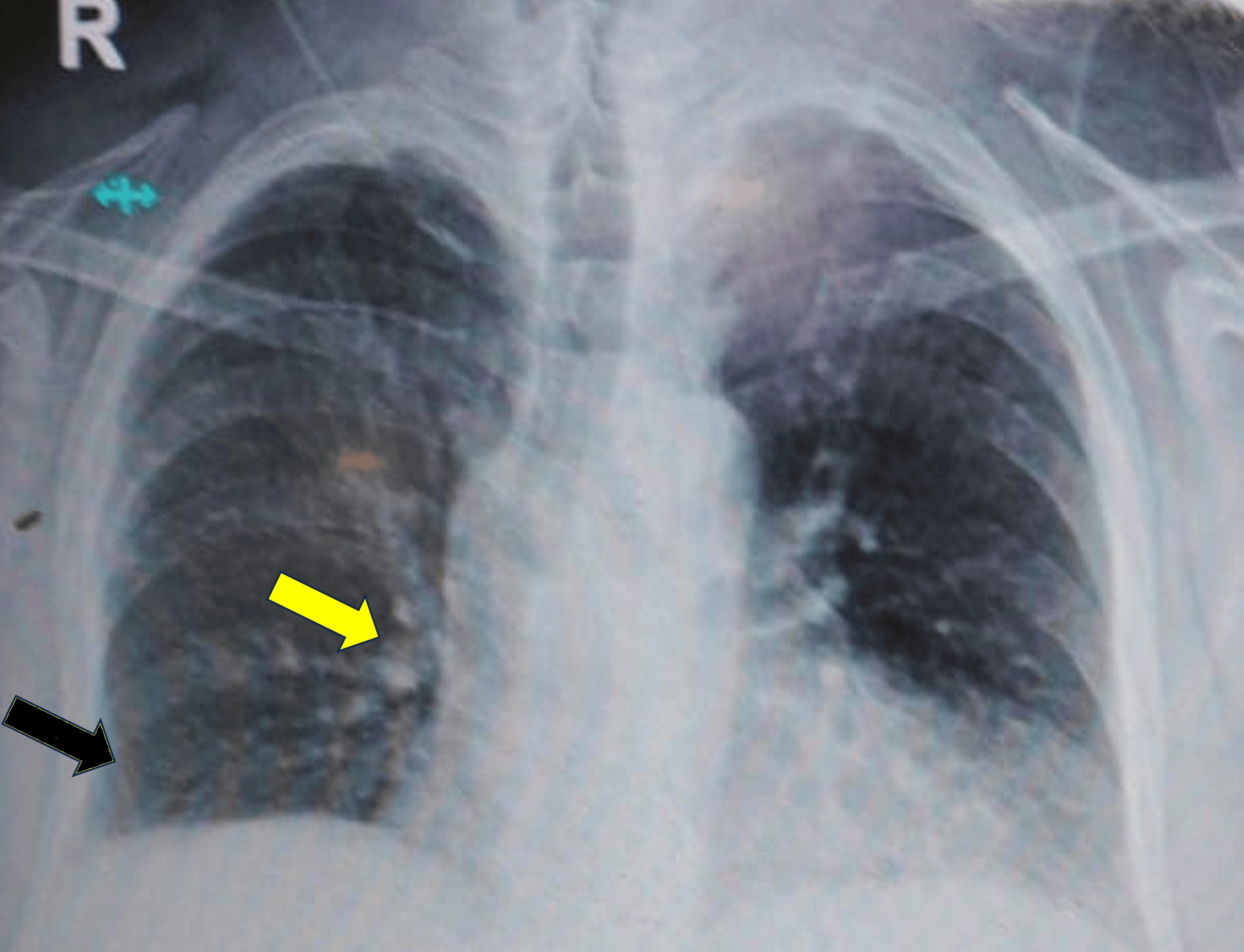
Chest X-ray showing minimal pleural effusion (black arrow) in the right basal zone and Palla's sign (yellow arrow), along with features of a lower respiratory tract infection Palla's sign: prominent enlargement of the right descending pulmonary artery.

Physical examination of the respiratory system demonstrated reduced air entry in the right basal zone, while other systemic examinations were within reference limits. Laboratory results showed alanine aminotransferase (85 U/L) and aspartate aminotransferase (140 U/L), both above reference ranges; NT-pro BNP was 582 µg/dL, which is slightly elevated but not significant enough to cause symptoms, while other parameters were within reference ranges (Table 1). A lower limb Doppler study showed no evidence of DVT. A chest X-ray (Figure 2) revealed significant abnormalities, including the Palla sign (enlargement of the right descending pulmonary artery) and minimal right pleural effusion, along with features consistent with a lower respiratory tract infection. High-resolution computed tomography of the thorax demonstrated minimal right pleural effusion with atelectasis.

**Table 1:** Laboratory investigations aPTT, activated partial thromboplastin time; ALT, alanine aminotransferase; AST, aspartate aminotransferase; INR, international normalized ratio; NT-proBNP, N-terminal pro-B-type natriuretic peptide

Analyte	Result	Reference Range
Hemoglobin	14.3 mg/dL	13-17 mg/dL
Total leukocyte count	10.3 × 10^3^/µL	4-11 × 10^3^/µL
Platelet count	315 × 10^3^/µL	150-400 × 10^3^/µL
Serum urea nitrogen	16 mg/dL	17-43 mg/dL
Creatinine	0.94 mg/dL	0.72-1.18 mg/dL
Sodium	137 mmol/L	136-146 mmol/L
Potassium	4.8 mmol/L	3.5-5.1 mmol/L
Chloride	105 mmol/L	101-109 mmol/L
Total bilirubin	0.74 mg/dL	0.3-1.2 mg/dL
Direct bilirubin	0.21 mg/dL	0-0.2 mg/dL
ALT	85 U/L	0-50 U/L
AST	140 U/L	0-50 U/L
Albumin	3.9 g/dL	3.5-5.2 g/dL
NT-proBNP	582 µg/dL	Up to 500 µg/dL
INR	1.5	0.8-1.1
aPTT	15 sec	21-35 sec
Fibrinogen	459 mg/dL	200-400 mg/dL
d-Dimer	357 µg/dL	80-500 µg/dL

**Figure 3:**
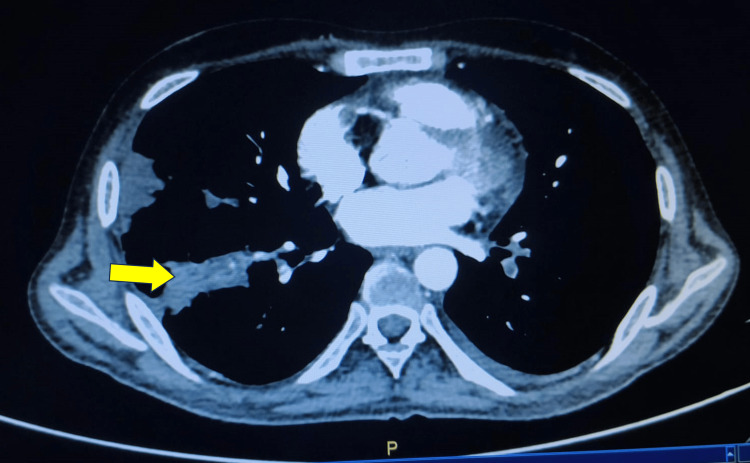
Computed tomographic pulmonary angiography demonstrating segmental thromboembolism in the right lower lobe pulmonary artery (yellow arrow)

The patient’s oxygen saturation remained low despite initial treatment based on differential diagnoses. Incentive spirometry improved the atelectasis and pleural effusion but did not normalize his oxygen saturation. Following the PE algorithm, the Wells score was <4, and the D-dimer was not significantly elevated; however, a high clinical suspicion persisted based on the PE Rule-out Criteria. Consequently, a computed tomographic pulmonary angiography confirmed thromboembolism in the segmental branches of the right lower lobe pulmonary artery (Figure 3). The patient was started on apixaban, showed clinical improvement, and was discharged in a hemodynamically stable state.

## Discussion

The most common cause of PE is DVT. In this case, however, the source of the embolism was unclear. A case report by Saboo et al. suggests that the coagulation cascade is altered at high altitudes. There is a transient hypercoagulable state in individuals who spend more than a week at high altitudes [2].

Kotwal et al. demonstrated that the combination of erythrocytosis, increased platelet count, platelet activation, and elevated fibrinogen level, combined with hypoxia and dehydration at high altitudes, creates a thrombotic milieu. This environment can lead to thrombosis in healthy people or in asymptomatic individuals with inherited or acquired thrombophilia [4].

Shlim et al. described a similar case in which a woman who had been taking oral contraceptive pills for many years developed pulmonary symptoms while climbing at a high altitude. She was later found to have a PE with left leg DVT. Before her ascent, she had never experienced any symptoms or signs of DVT, suggesting that high altitude exacerbated a hypercoagulable state [5].

Gupta et al. described how exposure to high-altitude environments, whether through air travel, mountain climbing, or participation in high-altitude sports, can induce a hypercoagulable state [6]. Several environmental factors at high altitudes, including hypoxia, dehydration, hemoconcentration, cold temperatures, and restrictive clothing, contribute to the development of thrombotic disorders. The three key components of Virchow’s triad, venous stasis, hypercoagulability, and vascular injury, are present in high-altitude environments, suggesting a complex interplay of factors. Given the numerous environmental variables involved, high-altitude-induced thromboembolic disorders likely result from multiple contributing factors rather than a single cause [6].

A large prospective longitudinal study by Nair et al. provided further evidence of thrombotic events occurring in acclimatized lowlanders exposed to high altitudes [7]. The study identified 15 individuals who experienced thrombotic events, including 12 venous and three arterial cases. Affected individuals exhibited significantly higher levels of procoagulant factors VIIa and Xa and lower levels of anticoagulant modulators thrombomodulin and tissue factor pathway inhibitors compared to healthy controls [7].

In our patient’s case, the absence of typical risk factors for PE and the acute onset of symptoms following a multiday ascent to elevations exceeding 3000 m strongly suggest that altitude-related physiological changes played a key role in developing pulmonary thromboembolism. His laboratory results, which did not reveal any clear thrombotic source such as DVT, reinforce the likelihood that hypoxia, dehydration, and other altitude-induced factors contributed to a transient hypercoagulable state. Moreover, the rapid improvement upon initiating anticoagulation highlights the need for prompt consideration of PE in patients presenting with unexplained hypoxia and respiratory distress at high altitudes, even in those without traditional risk factors.

## Conclusions

Pulmonary thromboembolism is a serious, life-threatening condition that can be fatal within minutes if not recognized promptly. PE is typically seen in patients with risk factors such as DVT, immobilization, recent surgery, or malignancy. However, rare cases have emerged in individuals at high altitudes, with or without these known risk factors. The exact mechanism of PE at high altitudes is not fully understood, although alterations in the coagulation cascade are considered a potential cause. Further studies on populations living at high altitudes are needed to investigate coagulation profiles and other contributing factors. A deeper understanding of these risks and underlying mechanisms could improve prevention and management strategies. Clinicians should maintain a broad differential diagnosis to include rare entities like high-altitude PE.
